# Plasminogen is a master regulator and a potential drug candidate for the healing of radiation wounds

**DOI:** 10.1038/s41419-020-2397-0

**Published:** 2020-03-23

**Authors:** Mahsa Fallah, Emil Viklund, Assar Bäckman, Jessica Brodén, Bertil Lundskog, Michael Johansson, Michael Blomquist, Malgorzata Wilczynska, Tor Ny

**Affiliations:** 1 Department of Medical Biochemistry and Biophysics, Umeå University, 901-87 Umeå, Sweden; 2 Omnio AB, Tvistevägen 48, 907-36 Umeå, Sweden; 3 Department of Medical Biosciences, Pathology, Umeå University, 901-87 Umeå, Sweden; 4 Department of Radiation Sciences, Umeå University, 901-87 Umeå, Sweden

**Keywords:** Proteases, Drug discovery, Signal transduction, Experimental models of disease

## Abstract

Around 95% of cancer patients undergoing radiotherapy experience cutaneous side effects, and some develop radiation wounds or fibrosis. Currently, there is no effective treatment for these indications. We show here that plasminogen administration enhanced the healing of radiation wounds via pleiotropic effects on gene expression. Using RNA sequencing, we found that plasminogen downregulated the expression of genes in the TLR, TNF, WNT, MAPK, and TGF-β signaling pathways, and enhanced the anti-inflammatory effect of arachidonic acid, leading to significantly decreased inflammation and improved remodeling of granulation tissue compared with placebo treatment. In addition, plasminogen induced metabolic changes, including decreased glycolysis. Importantly, many of the factors downregulated by plasminogen are pro-fibrotic. Therefore, in radiation wounds with excessive inflammation, plasminogen is able to enhance and redirect the healing process, such that it more closely resembles physiological healing with significantly reduced risk for developing fibrosis. This makes plasminogen an attractive drug candidate for the treatment of radiation wounds in cancer patients.

## Introduction

Ionizing radiation is an important treatment tool for various tumors, but it often has dose-limiting pathological effects on noncancerous “normal” tissue^1–4^. The sensitivity of tissues to irradiation depends on the number of dividing cells. Therefore, normal tissues with a relatively high proliferative capacity, such as the skin, are more severely affected by radiotherapy^1^. Radiation skin injuries are divided into acute and late injuries. Acute injuries appear within hours to weeks after irradiation, and include skin erythema, edema, desquamation, and/or ulcers. In fact, most patients undergoing radiotherapy develop some type of acute skin reaction. Late side effects of irradiation, such as ulceration or fibrosis, can develop months to years after the treatment^2^.

Dermal wounds are normally caused by rapid mechanical, chemical, or thermal trauma, and the healing for these wounds consists of partially overlapping phases of inflammation, tissue formation, and tissue remodeling. Under physiological conditions, each of the healing phases is properly activated and terminated through the coordination of a large number of signaling molecules, including cytokines, chemokines, and growth factors. Abnormal activation of any of these phases, or their lack of termination, results in the formation of chronic wounds^5^. In contrast, radiation wounds develop over time as a consequence of a burst of inflammation evoked by radiation-induced oxidative stress, and a deficient tissue turnover caused by damage to stem cells^3,4,6^. Therefore, by the time the radiation wounds appear, they are characterized by an already abnormally high inflammation and chronic overproduction of specific cytokines^7^. Irradiation aberrantly activates various cell-signaling pathways that disrupt the normal wound-healing process. Also, tissue levels of various cytokines, chemokines, and growth factors involved in normal inflammatory reactions in acute wounds, such as interleukin 1β (IL1-β), interleukin 6 (IL-6), vascular endothelial growth factor (VEGF), transforming growth factor-β (TGF-β), and tumor necrosis factor-α (TNF-α), are dysregulated in radiation wounds^7–9^. In addition, high levels of reactive oxygen species (ROS) generated by the radiation impair neovascularization, the formation of granulation tissue, and re-epithelialization^7^. Moreover, fibroblasts, which play an important role in the remodeling phase of acute wounds, produce a highly disorganized ECM in radiation wounds, which leads to reduced wound strength and fibrosis^10^.

The plasminogen activator system is a temporally controlled proteolytic system in which plasminogen is activated to plasmin by either tissue-type or urokinase-type plasminogen activator. This system, initially identified as a fibrinolytic system, is also involved in wound healing^11^. Generally, during the wound-healing process, a plasminogen activator produced by different types of cells locally converts plasminogen to plasmin. Early studies in plasminogen-deficient mice showed impaired wound healing due to delayed keratinocyte migration^12^. To date, several functions have been assigned to plasmin in wound healing, including dissolving fibrin, degrading the ECM, activating growth factors, and promoting angiogenesis^13^. We have previously shown that plasminogen plays a regulatory role in the initiation and termination of the inflammatory phase during wound healing, and that plasminogen supplementation restores the healing of acute wounds in plasminogen-deficient mice, and enhances healing in both wild-type and db/db diabetic mice^14–16^.

In this study, we show that administration of plasminogen accelerates the healing of radiation wounds in wild-type mice. Transcriptome analysis revealed that plasminogen corrected the expression levels of many genes that are dysregulated in radiation-induced wounds to levels seen in nonirradiated skin. This was accompanied by improved healing quality and accelerated resolution of inflammation in plasminogen-treated wounds. This pleiotropic regulatory effect of plasminogen administration in radiation wounds makes plasminogen a potential new drug candidate for the treatment of radiation-induced skin wounds in cancer patients.

## Results

### Plasminogen accelerates the healing of radiation-induced wounds

We have previously shown that plasminogen accumulates in burn wounds in wild-type mice, and activates an inflammatory response during the healing process^14^. In mice where accumulation of plasminogen is insufficient, as it is in db/db diabetic mice, or in plasminogen-deficient mice, the wound healing is delayed and aberrant^14–16^. Importantly, plasminogen supplementation restores the healing of burn wounds in plasminogen-deficient mice, and accelerates the healing process in wild-type and db/db diabetic mice^14,15^.

**Fig. 1:**
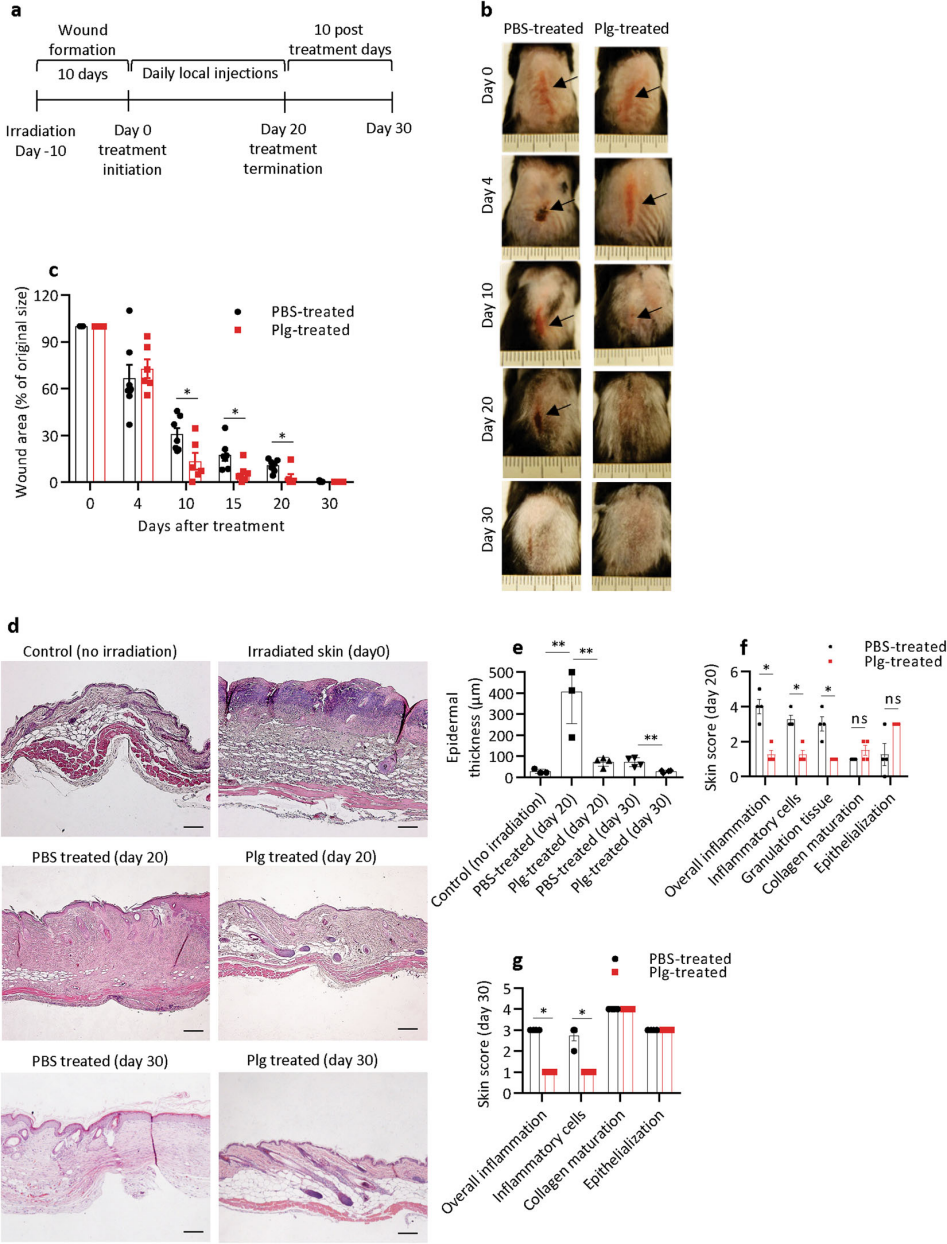
Plasminogen accelerates and improves the quality of healing in radiation wounds. **a** The timescale for radiation wound formation and treatment. **b** Representative photographs of the dorsal skin of wild-type mice at day 0 (before treatment) and at different time points with plasminogen or PBS treatment. Arrows show the wounds on the skin. **c** Quantification of the remaining wound area at different time points of treatment in the PBS-treated wounds (*n* = 6) and plasminogen-treated wounds (*n* = 6). **d** Representative photographs of hematoxylin and eosin-stained sections of normal/healthy skin, and sections of radiation wounds at day 0 before treatment, and at days 20 and 30 of PBS or plasminogen treatment. **e** Epidermal thickness of healthy skin (control, *n* = 3) and thickness of the epidermis in radiation wounds treated with plasminogen (*n* ≥ 3) or PBS (*n* ≥ 3) at days 20 and 30 of treatment. **f**, **g** Characterization of the healing quality of wounds treated with PBS or plasminogen at days 20 and 30, respectively, performed according to the scoring system presented in Supplementary Table 1. *P < 0.05, ** *P* < 0.01; ns not significant (*P* > 0.05); plg plasminogen. Scale bar = 100 µm.

The healing of radiotherapy-induced cutaneous wounds in cancer patients is often suppressed^10,17^. We therefore investigated whether local administration of plasminogen could also enhance the healing of radiation wounds. The dorsal skin of wild-type mice was locally irradiated^6^ with a single dose of 20 Gy of ionizing radiation, and the mice were then left untreated for 10 days to develop radiation wounds in the form of desquamation^6^. At day 10 after irradiation, defined here as day 0 of treatment (Fig. 1a), the mice were divided into two groups: one group received daily subcutaneous injections of plasminogen, whereas the other group (control) received injections of placebo (PBS). The injections were performed for 20 consecutive days, and then the mice were left untreated for the next 10 days before being sacrificed at day 30 (Fig. 1a). Initially, the wound area remained similar in both groups; however, from day 10 to 20, the wounds in the plasminogen-treated mice were significantly smaller than in the control mice (Fig. 1b, c). During the additional 10 post-treatment days, all wounds in both groups were completely re-epithelialized. Skin samples taken at days 20 and 30 were embedded in paraffin, sectioned, and stained with Mayer’s hematoxylin. At day 20, the epidermis in plasminogen-treated mice was only about twice as thick as nonirradiated skin, whereas the epidermis in PBS-treated wounds was 15-fold thicker (Fig. 1d, e). At day 30, the thickness of the epidermis in plasminogen-treated wounds was as in healthy skin, but the epidermis in PBS-treated wounds was still 2.6-fold thicker. To further assess the histopathology of the skin, we used a scoring system (Supplementary Table 1). During normal wound healing, inflammation and granulation tissue should diminish as the healing progresses, while collagen maturation and epithelialization should increase over time. As shown in Fig. 1d, f, significantly lower overall inflammation, less inflammatory cell infiltration, and less granulation tissue were observed in the plasminogen-treated mice at day 20 compared with mice treated with PBS. In addition, epithelialization appeared more advanced in plasminogen-treated wounds. At day 30, despite complete wound closure in both treatment groups, the post-wounded area in PBS-treated mice had epithelial hyperplasia and higher inflammation compared with plasminogen-treated mice (Fig. 1d, g). This shows that plasminogen administration to radiation-induced wounds significantly enhanced the healing rate and improved the healing quality of radiation wounds.

### Plasminogen reduces the accumulation of fibrin and neutrophils during the healing of radiation wounds

**Fig. 2:**
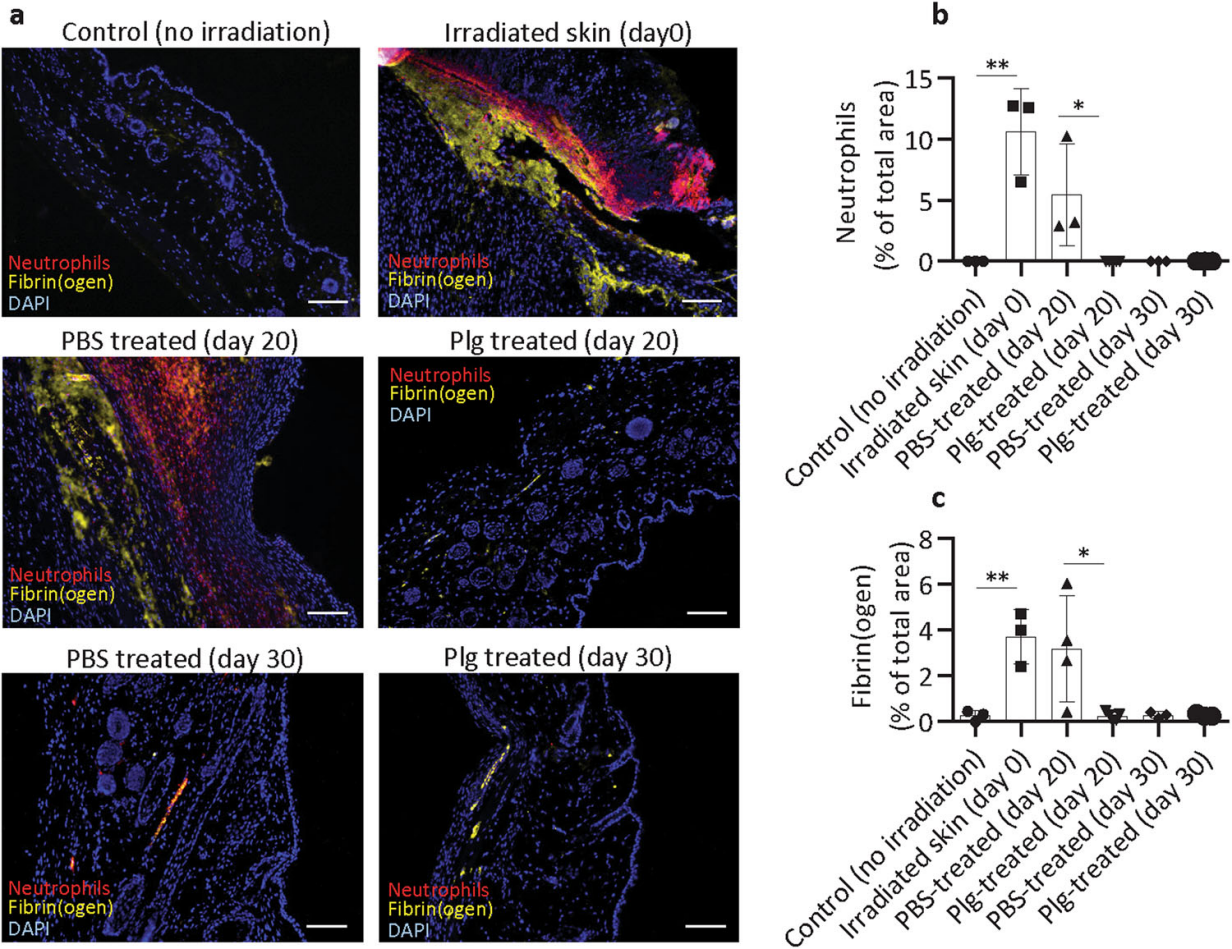
Plasminogen treatment reduces the accumulation of fibrin and neutrophils in radiation wounds. **a** Immunostaining of fibrin (yellow) and neutrophils (red) performed on paraffin sections from healthy skin (no irradiation), skin at 10 days after irradiation (day 0), and radiation wounds treated with PBS or plasminogen at days 20 and 30. DAPI staining is shown in blue. **b**, **c** Quantification of neutrophils and fibrin (respectively) in healthy skin, non-treated radiated skin (day 0), and in PBS- and plasminogen-treated radiation wounds at days 20 and 30 (*n* ≥ 3). *P < 0.05, **P < 0.01; scale bar = 100 µm.

We have previously shown that treatment of burn wounds with plasminogen leads to faster and better healing due to accelerated removal of neutrophils and fibrin from the wounded area^15^. Here, we show that extravascular neutrophils and fibrin deposits are absent in healthy, nonirradiated skin^6^, but excessive accumulation of fibrin and neutrophils was observed in radiation-induced wounds at day 0 (before treatment) and remained high in wounds treated with PBS for 20 days (Fig. 2a–c). In contrast, at day 20 of plasminogen treatment, only a residual amount of fibrin was detected, and neutrophils were mostly absent in the wounded area. This shows that plasminogen administration to radiation wounds accelerated the resolution of inflammation and tissue debridement compared with PBS treatment.

### Radiation wounds are characterized by high expression of genes that regulate inflammation and tissue formation

To investigate the molecular events that take place during the healing of radiation wounds, we studied the expression of genes involved in wound healing using the Mouse Wound Healing RT^2^ Profiler PCR array.

**Fig. 3:**
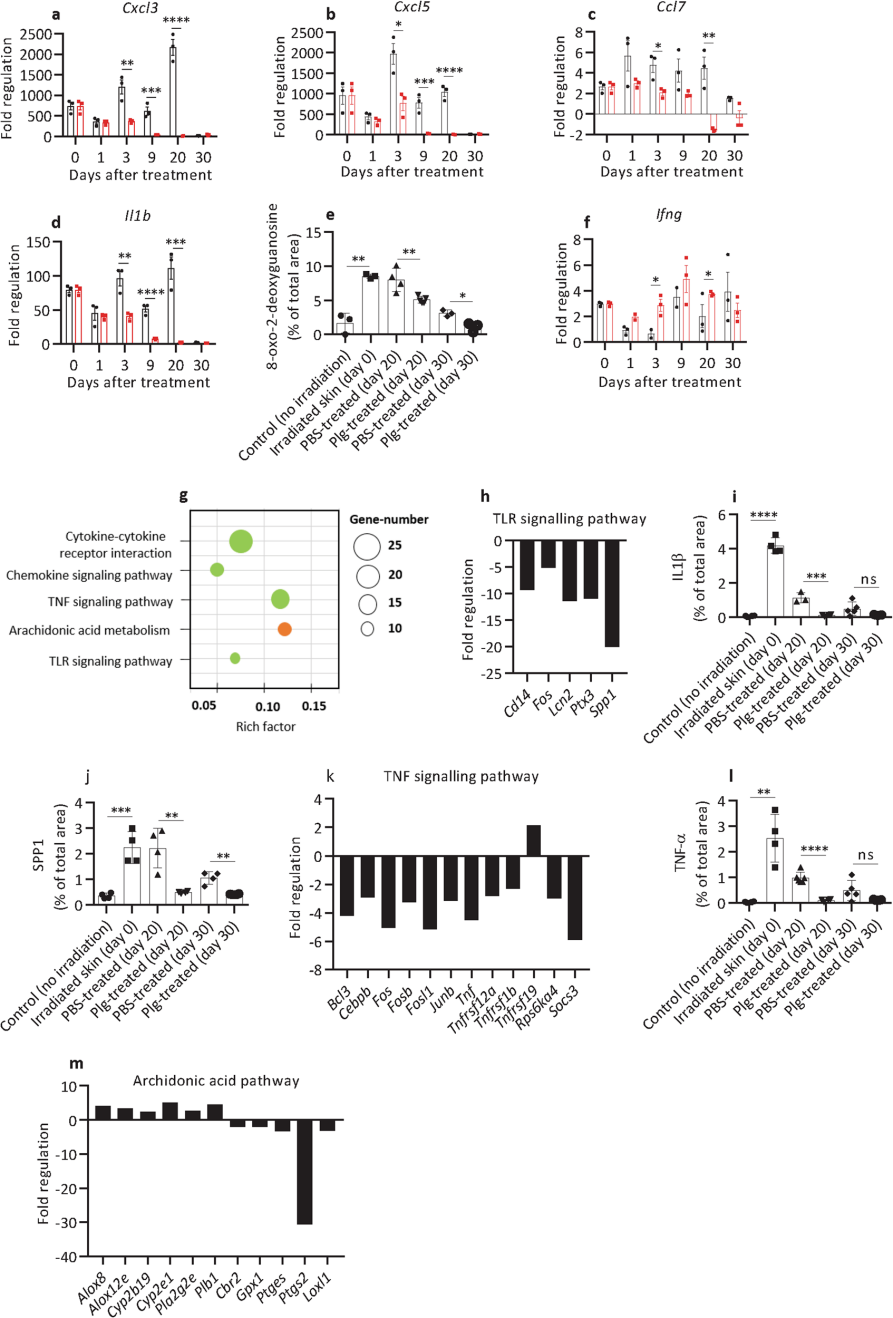
Plasminogen treatment of radiation wounds results in the decreased expression of genes involved in inflammation. **a**–**d**, **f** Expression of selected inflammatory genes (as indicated) in radiation wounds before treatment (day 0) (*n* = 3) and at different time points of PBS (black) (*n* = 3) or plasminogen (red) (*n* = 3) treatment as measured with the Mouse Wound Healing RT^2^ Profiler PCR array. The mRNA levels for all samples are presented as fold regulations relative to the mRNA levels in the control healthy, nonirradiated skin. (**e**) Quantification of 8-oxo-2-deoxyguanidine in healthy non-radiated skin (*n* = 3), radiated skin before treatment (day 0) (*n* = 3), and in PBS- (*n* ≥ 3) and plasminogen-treated (*n* ≥ 3) radiation wounds at days 20 and 30. **g** The KEGG enrichment scatter plot of inflammatory genes/pathways that were differentially expressed in plasminogen-treated wounds at day 20 when compared with PBS-treated wounds, as identified using mRNA sequencing. Downregulated pathways are shown in green, and the pathway that includes both upregulated and downregulated genes is shown in orange. **h** Expression of genes in the TLR pathway that were significantly changed at day 20 of plasminogen treatment when compared with PBS treatment, as measured using mRNA sequencing. **i**, **j** Quantification of protein levels for IL1-β and Spp1 (respectively) in healthy non-radiated skin, irradiated skin before treatment (day 0), and in PBS- and plasminogen-treated radiation wounds, based on immunostaining of paraffin sections (*n* ≥ 3). **k** Expression of genes in the TNF pathway that were significantly changed at day 20 of plasminogen treatment when compared with PBS treatment, as measured using mRNA sequencing. **l** Quantification of TNF-α protein levels as described in panel **i** (*n* ≥ 3). **m** Expression of genes in the arachidonic acid pathways that were significantly changed at day 20 of plasminogen treatment when compared with PBS treatment, as measured using mRNA sequencing. *P < 0.05; **P < 0.01; ***P < 0.005; ****P < 0.001. Rich factor is the ratio of the number of target genes divided by the number of all the genes in each pathway.

In radiation wounds at day 0 (Supplementary Table 2), the expression of 33 genes out of the 84 genes studied was significantly changed, compared with healthy nonirradiated skin. Radiation wounds had high expression of pro-inflammatory chemokines (*Cxcl3*, *Cxcl5*, *Cxcl1*, and *Ccl7*), interleukins (*Il1b*, *Il6*, and *Il4*), Cd40 ligand (*Cd40lg*), and prostaglandin synthase 2 (*Ptgs2*) (Supplementary Table 2, Fig. 3a–d, day 0). In addition, high expression of ECM-remodeling factors (*Mmp9*, *Plaur*, *Plat*, *Serpine1*, and *Timp1*), cell adhesion molecules (*Itga1*, *Itga3*, *Itga4*, *Itga5*, *Itga6*, and *Itgav*), and regulators of actin stress fibrils (*Rhoa* and *Tagln*) indicated an extensive cell migration and tissue remodeling in the wounded area. This was paralleled by the elevated expression of growth factors that stimulate cell proliferation and neoangiogenesis (*Csf3*, *Hbegf*, *Hgf*, and *Vegfa*), as well as activation of TGF-β signaling (enhanced expression of *Tgfb1* and *Stat3*) and WNT signaling (enhanced expression of *Wisp1* and *Wnt5*). The only two genes downregulated in these wounds were the cytoskeleton actins *Acta2* and *Actc1*. Because Actc1 is expressed in myofibroblasts^18^, its reduced expression might suggest insufficient fibroblast activation in this stage of radiation wounds. These results are in line with previous reports describing high levels of inflammation and aberrant tissue formation in wounds induced by ionizing radiation^8,10^.

### Plasminogen treatment of radiation-induced wounds results in a decreased expression of pro-inflammatory genes

The effect of plasminogen on gene expression during the healing of radiation wounds was initially determined using the Mouse Wound Healing and Fibrosis RT^2^ profiler PCR arrays (Supplementary Table 3). In general, plasminogen treatment resulted in downregulation of genes that were upregulated in radiation wounds, and in upregulation of genes that were suppressed before the treatment (genes marked by an asterisk in Supplementary Table 3). Therefore, plasminogen seems to return gene expression to about the levels observed in healthy nonirradiated skin. In contrast, at day 20 of PBS treatment, gene expression in radiation-induced wounds remained highly dysregulated.

As shown in Fig. 3a–d, Supplementary Table 3, and Supplementary Fig. 1, during the 20 days of PBS treatment, the expression of pro-inflammatory genes remained at the same high levels as before the treatment, or increased even further. In contrast, in plasminogen-treated wounds, the expression of inflammatory genes started to decline from day 1 and decreased gradually over the 20 days of the treatment. The inflammatory genes included *Tnf1* (a major regulator of acute inflammation^19^), *Ptgs2* (prostaglandin synthase), and chemoattractants for neutrophils (*Cxcl1*, *Cxcl3*, *Cxcl5*, and *IL1b*) and monocytes (*Mif*, *Ccl7*, and *IL-6*)^20^. Therefore, plasminogen treatment diminished the migration of immune cells into the wounded area (see also Fig. 2a, b). Low inflammation in plasminogen-treated wounds correlated with low levels of ROS, as shown by 8-oxo-2-deoxyguanidine staining (Fig. 3e and Supplementary Fig. 2). Even though expression of the pro-inflammatory genes studied had decreased in PBS-treated wounds at day 30, the level of ROS was still higher as compared with plasminogen-treated wounds.

The only cytokine that was transiently upregulated by plasminogen was *Ifng* (Fig. 3f). Ifng is an important activator of phagocytic activity of macrophages^21^, and is therefore important for the proper resolution of inflammation^22,23^. It is likely that the induction of *Ifng* is linked with the plasminogen-induced wound debridement described by us previously^15^.

To further elucidate the molecular mechanisms behind the healing effect of plasminogen, we performed mRNA sequencing of the samples taken at day 20 of treatment. This study confirmed a pleiotropic effect of plasminogen (Fig. 3g), and showed that plasminogen treatment decreased the expression of many pro-inflammatory factors, including cytokines, interleukins, and their receptors (Supplementary Table 4). This might at least partially be caused by plasminogen-dependent inhibition of the TLR and TNF signaling pathways (Fig. 3g–l, and Supplementary Figs. 3–5). The only inflammation-related genes upregulated by plasminogen were *Cxcl9* (an anti-angiogenic and anti-fibrotic factor)^24^ and *Tnfrf19* (an inhibitor of TGF-β signaling)^25^.

Inflammation is also regulated by the arachidonic acid metabolic pathway. Arachidonic acid is released from membrane phospholipids by phospholipases, and can be converted into pro-inflammatory prostaglandins via specific prostaglandin synthases and oxidases. Alternatively, it can be metabolized by Cyp (cytochrome P450) enzymes and lipoxygenases to anti-inflammatory hydroxyeicosatetraenoic acids (HETEs) and epoxyeicosatrienoic acids (EETs)^26,27^. As shown in Fig. 3m and Supplementary Table 4, at day 20 of treatment, plasminogen upregulated the expression of phospholipases (*Pla2g2e* and *Plb1*) and the enzymes involved in HETE and EET synthesis (*Alox8*, *Alox12e*, *Cyp2b19*, and *Cyp2e1*), and simultaneously decreased the expression of enzymes involved in the prostaglandin pathway (*Ptges*, *Ptgs2*, *Cbr2*, *Gpx1*, and *Loxl1*).

Taken together, these results suggest that plasminogen treatment of radiation wounds downregulates pro-inflammatory signaling pathways and upregulates anti-inflammatory pathways.

### Plasminogen regulates the expression of genes responsible for granulation tissue formation and remodeling

For wounds to heal, the granulation tissue formed during the tissue formation phase has to be resolved. If granulation tissue is not removed in time, it will impede the healing process, and lead to the development of chronic wounds and fibrosis^28^. The formation and resolution of granulation tissue are synchronized by transiently activated cell-signaling pathways that regulate the expression of growth factors and other genes involved in cell proliferation and migration, angiogenesis, and remodeling of the ECM^29^.

**Fig. 4:**
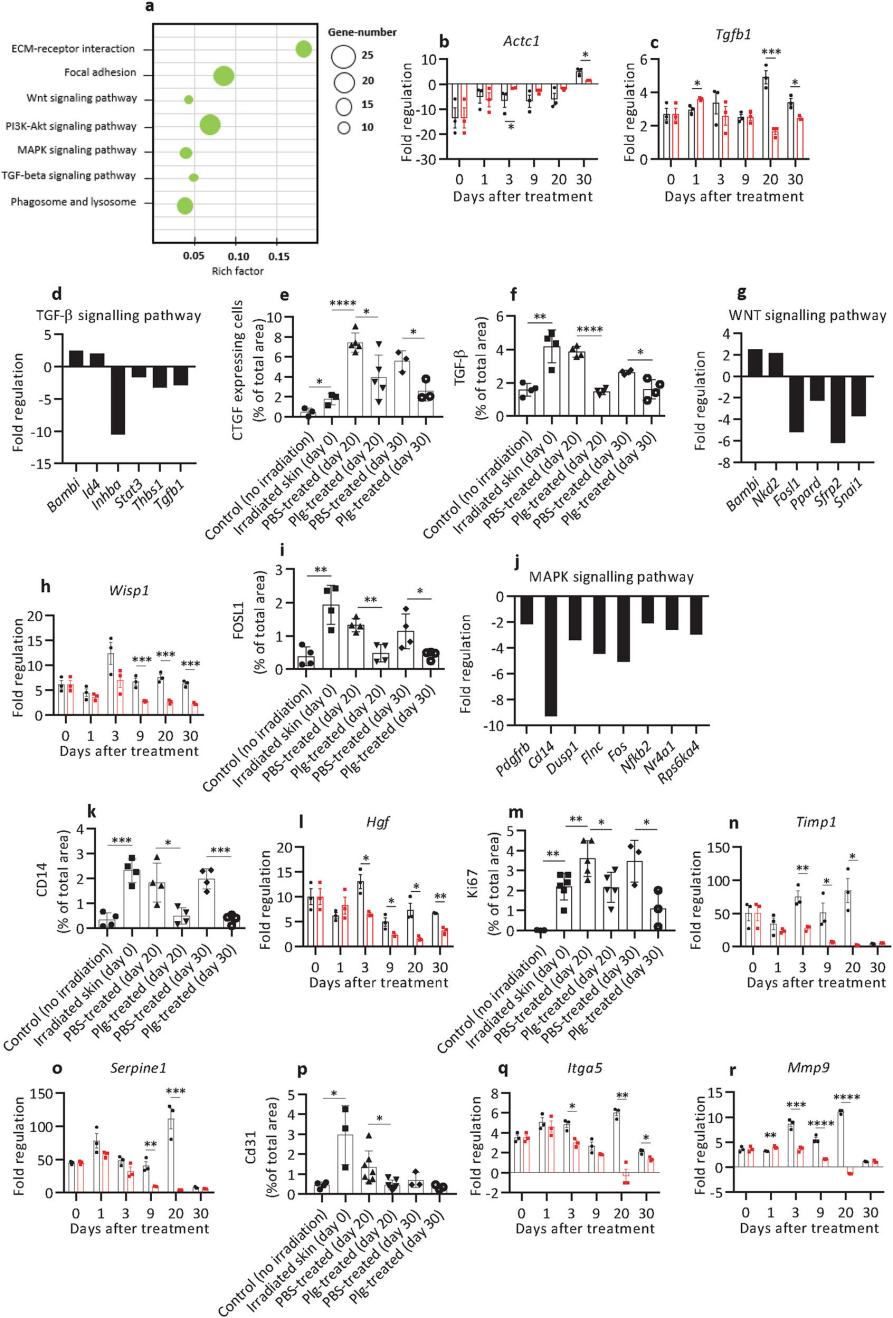
Plasminogen treatment regulates the expression of genes responsible for the formation and remodeling of granulation tissue. **a** The KEGG enrichment scatter plot of genes involved in the formation and remodeling of granulation tissue that were significantly downregulated in plasminogen-treated wounds at day 20 when compared with PBS-treated wounds, as identified using mRNA sequencing. **b**, **c** Expression of *Actc1* and *Tgfb1* (respectively) in radiation wounds before treatment (day 0) (*n* = 3) and at different time points of PBS (black) (*n* = 3) or plasminogen (red) (*n* = 3) treatment as measured with the Mouse Wound Healing RT^2^ Profiler PCR array. The mRNA levels for all samples are presented as fold regulation relative to mRNA levels in the control healthy, nonirradiated skin. **d** The expression of genes involved in the TGF-β pathway that were significantly changed in plasminogen-treated wounds at day 20 when compared with PBS-treated wounds, as measured using mRNA sequencing. **e**, **f** Quantification of CTGF and TGF-β (respectively) protein levels in healthy non-radiated skin, in irradiated skin before treatment (day 0), and in PBS- and plasminogen-treated radiation wounds at days 20 and 30, based on immunostaining of paraffin sections from the wounded area (*n* ≥ 3). **g** Expression of genes involved in the WNT pathway measured as described in panel **d**. **h** Expression of Wisp1 in radiation wounds shown as described in panel **b**. **i** Quantification of FOSL1 protein levels as described in panel **e** (*n* ≥ 3). **j** Fold regulation of MAPK pathway-related genes that were significantly enriched at day 20 of plasminogen- compared with PBS treatment. **k** Quantification of CD14 protein levels as described in panel **e** (*n* ≥ 3). **l** Expression of *Hgf* in radiation wounds measured as described in panel **b**. **m** Quantification of Ki67 protein levels as described in panel **e** (n ≥ 3). **n**, **o** Expression of *Timp1* and *Serpine1* in radiation wounds, respectively, shown as described in panel **b**. **p** Quantification of CD31 protein levels as described in panel **e** (n ≥ 3). **q**, **r** Expression of *Itga5* and *Mmp9* (respectively) in radiation wounds, shown as described in panel **b**. *P < 0.05; **P < 0.01; ***P < 0.005; ****P < 0.001. Rich factor is the ratio of the number of target genes divided by the number of all the genes in each pathway.

Plasminogen treatment affected the expression of many genes involved in the remodeling of granulation tissue (Fig. 4a, and Supplementary Tables 3 and 4). As shown in Fig. 4b and Supplementary Table 3, from day 3 to day 20, the expression of *Actc1* was enhanced, suggesting that plasminogen transiently stimulated the development of myofibroblasts. At late time points of wound healing, plasminogen downregulated the expression of *Tgfb1* (Fig. 4c) and inhibited TGF-β signaling via enhanced expression of inhibitors (*Bambi* and *Id4*), and decreased expression of several effector genes in this pathway (Fig. 4d). In line with these data, the protein levels of TGF-β-induced connective tissue growth factor (CTGF) and TGF-β in wounds treated by plasminogen were significantly lower than in PBS-treated wounds (Fig. 4e, f, and Supplementary Figs. 6 and 7). Plasminogen also inhibited the WNT signaling pathway by increasing the expression of WNT pathway inhibitors (*Bambi* and *NDK2*), and decreasing the expression of downstream effectors (Fig. 4g–i and Supplementary Fig. 8). In addition, plasminogen also suppressed the MAPK signaling pathway (Fig. 4j and k and Supplementary Fig. 9). This was associated with decreased expression of several growth factors involved in cell proliferation, such as *Fgf7*, *Fgf10*, *Hbegf*, *PdgfA*, *Tnf*, and *Hgf* (Fig. 4l and Supplementary Table 3) and decreased cell proliferation (shown by decreased expression of Ki67, Fig. 4m and Supplementary Fig. 10). These expression data are in line with a reduced volume of granulation tissue in plasminogen- compared with PBS-treated wounds (Fig. 1f).

Plasminogen treatment also decreased the expression of pro-angiogenic growth factors (*Csf3* and *Vegfa*^30–32^) and pro-angiogenic genes such as laminins, *Thbs1*, *cPla*, *Ptgs2*, *Timp1*, and *Serpine1* (Fig. 4n, o, Supplementary Tables 3 and 4). As a result, the number of vessels (shown by CD31 staining) at day 20 of treatment was significantly lower in plasminogen- than in PBS-treated wounds (Fig. 4p and Supplementary Fig. 11).

Phagocytosis is important for the resolution of inflammation, and for the removal of excessive fibroblasts and ECM components during the remodeling of granulation tissue^23,33^. As shown in Fig. 4a and Supplementary Table 4, at day 20, plasminogen treatment resulted in downregulation of nine genes involved in phagosome and lysosomal degradation, and four genes regulating the actin cytoskeleton, which suggests that the healing process was complete, and ECM remodeling and tissue debridement were no longer required.

### Plasminogen is a regulator of ECM composition during the healing of radiation wounds

The ECM is responsible for cell attachment, cell-to-cell communication, and often for cell activation^34^. The ECM thus plays an integral role in the entire wound repair process, and its content and volume change during the wound-healing process. Abnormally reconstituted ECM contributes to the formation of hypertrophic and keloid scars and fibrosis^35^.

Here we show that plasminogen treatment downregulated the expression of 22 ECM components during wound healing, including the expression of seven collagens, laminin-5, thrombospondin, osteopontin, and their integrin receptors (Fig. 4q, Supplementary Tables 3 and 4). The only ECM component that was upregulated by plasminogen was collagen 1, the major ECM component in healthy skin^35^. In parallel with the reduction of ECM components, plasminogen also reduced the expression of several ECM-remodeling enzymes (including seven MMPs and two plasminogen activators) and their inhibitors (Fig. 4n, o, and r, and Supplementary Tables 3 and 4).

### Plasminogen decreases the expression of pro-fibrotic factors

Radiation-induced fibrosis normally appears about 4–12 months after radiotherapym and can affect almost all parts of the body that were exposed to the radiation^36^. Fibrosis develops due to high levels of inflammation and ROS, and enhanced TGF-β signaling^37,38^ that leads to excessive deposition of ECM.

**Fig. 5:**
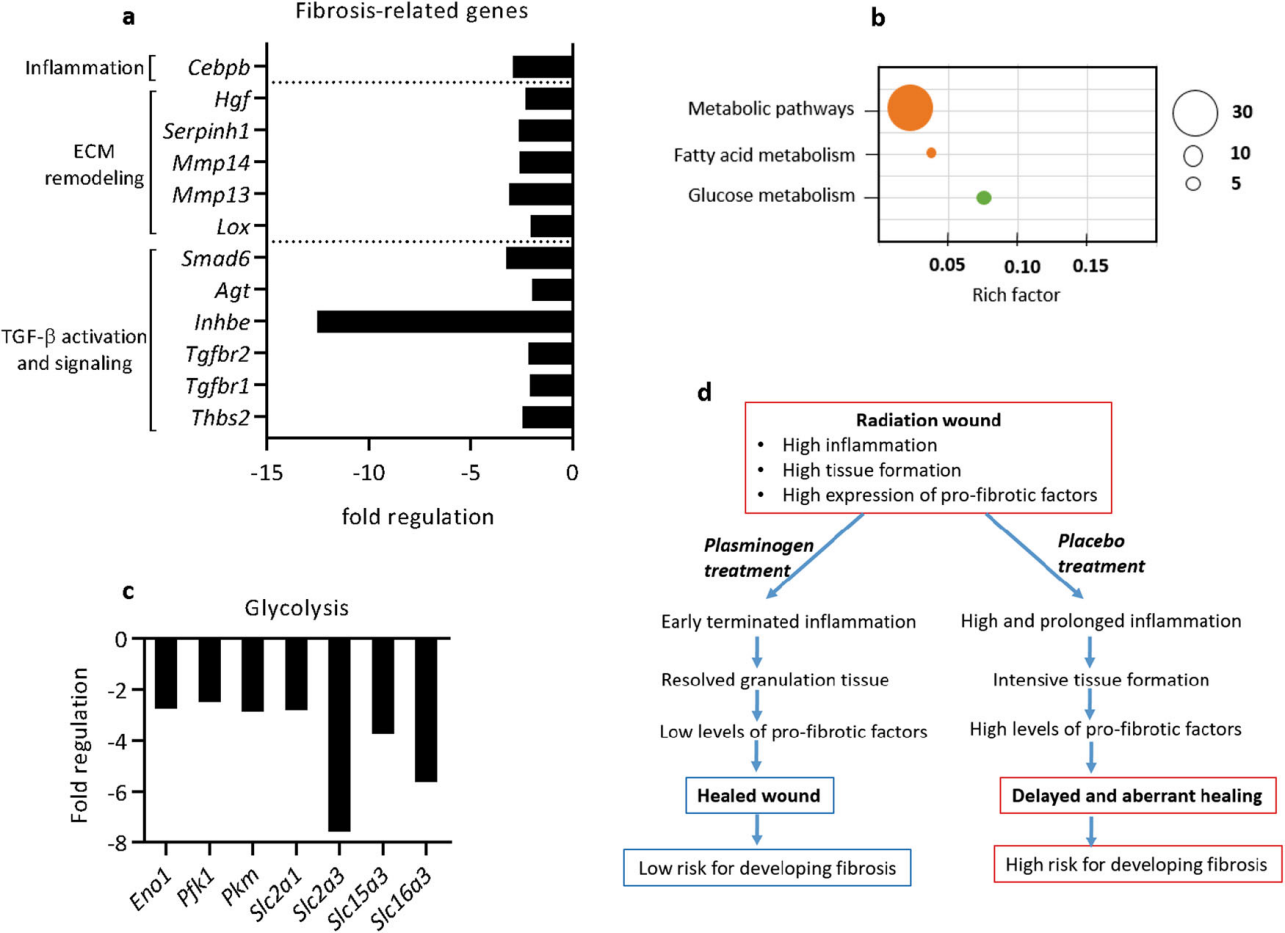
Plasminogen treatment leads to decreased expression of factors that are involved in the development of fibrosis. **a** Expression of genes that are involved in fibrosis that were significantly changed at day 20 of plasminogen treatment when compared with PBS treatment, as measured using mRNA sequencing. Rich factor is the ratio of the number of target genes divided by the number of all the genes in each pathway. **b** The KEGG enrichment scatter plot of genes regulating metabolism that were significantly changed at day 20 of plasminogen treatment compared with PBS treatment, as identified by mRNA sequencing. Downregulated genes are shown in green, and pathways that include both upregulated and downregulated genes are shown in orange. **c** Expression of genes involved in the regulation of glycolysis that were significantly changed in plasminogen-treated wounds at day 20 when compared with PBS-treated wounds, as measured using mRNA sequencing. **d** A model for the molecular mechanisms by which plasminogen enhances the healing of radiation wounds.

As shown in Fig. 5a, Supplementary Table 3 (genes marked by #), and Supplementary Table 4, at day 30, when all wounds were healed, plasminogen-treated mice had significantly lower expression of genes related to TGF-β activation (*Thbs2*) and TGF-β signaling (*Tgfbr 1/2*, *Inhbe*, and *Agt*). In addition, the expression of factors involved in ECM remodeling (*Mmp 13*, *Mmp14*, *Lox*, and *Serpinh1*), and in the activation of the epidermal-to-mesenchymal transition (*Hgf*), was lower in the post-wounded area in mice treated with plasminogen compared with the post-wounded area in mice treated with PBS. Plasminogen-treated wounds also had lower expression of *Smad6*, which is an inhibitor of anti-fibrotic ALK-3 signaling^39^. Finally, as shown in Fig. 4b, the level of *Actc1* (a marker of myofibroblasts) was significantly increased in PBS-treated mice at day 30, whereas it was at a normal physiological level in plasminogen-treated mice. The gene expression data were supported by histological experiments. The low protein level of CTGF and TGF-β in the post-wounded area in plasminogen-treated mice at day 30 (Fig. 4e, f) confirmed the inhibition of TGF-β signaling by plasminogen. In addition, wounds treated with plasminogen had lower levels of oxidative DNA damage (Fig. 3e), and lower levels of cell proliferation (Fig. 4m) at day 30, than wounds treated with PBS.

Fibrotic fibroblasts producing large amounts of ECM primarily use glycolysis as a source of energy, and fatty acid oxidation is downregulated in these cells. Suppression of glycolysis or induction of fatty acid oxidation in these fibroblasts inhibits the transcription of ECM genes, and changes the fibroblasts to a non-fibrotic phenotype^40^. Here we show that plasminogen treatment affected the expression of many genes that are involved in the regulation of tissue metabolism (Fig. 5b and Supplementary Table 4). In particular, the expression of genes involved in glycolysis was significantly suppressed in plasminogen-treated wounds already at day 20 (Fig. 5c). This strongly supports our finding that, in contrast to PBS-treated wounds, the highly energy-demanding cellular events of wound healing (inflammation, tissue formation, and remodeling) are fully completed, and fibrosis does not develop in plasminogen-treated wounds.

## Discussion

Skin injuries and fibrosis are the side effects of ionizing radiation that pose a significant problem for cancer patients undergoing radiotherapy. Despite many studies on the healing of radiation-induced wounds and the development of fibrosis, the molecular mechanisms behind these processes have not been fully elucidated. Moreover, no effective therapy exists in clinical practice to treat these radiation-induced side effects. Here we show that plasminogen accelerates the healing of radiation wounds (Fig. 1). Plasminogen administration to radiation wounds has a pleiotropic effect on gene expression that reflects the multiple roles of plasminogen in the wound-healing process. By suppressing inflammation and excessive granulation tissue formation, plasminogen induces a wound- healing process that has reduced risk for the development of fibrosis.

One of the major differences between radiation wounds and other types of cutaneous wounds (such as burns or cuts) is the timing of inflammation and granulation tissue formation. Radiation wounds are initiated by ROS and inflammatory burst caused by the radiation, but the wound itself only appears several days after the irradiation^6,10,41^. Therefore, at the time when radiation wounds are formed, the processes of inflammation and granulation tissue formation are already largely activated. In the other types of cutaneous wounds, inflammation is only activated after the wounding takes place, and granulation tissue is formed a few days later^23^. We have previously shown that plasminogen administrated to burn wounds has a pro-inflammatory effect^14,15,42^, but here we show that plasminogen administered to radiation wounds directly ameliorates inflammation. This discrepancy can most likely be explained by the fact that plasminogen has over 11 receptors that facilitate plasminogen activation and that are expressed on different cells. Regulated expression of these receptors provides spatial and temporal plasmin activity, depending on the processes being carried out in the tissues^43–45^. Therefore, by binding to these differentially expressed receptors, plasminogen might “sense” the environmental need for proteolytic activity, and thus activate inflammation in burn wounds, but reduce excessive inflammation in radiation wounds.

To date, several different mechanisms for the delayed healing of radiation wounds have been proposed, including excessive inflammation, an imbalance of cytokines and growth factors, ECM alterations, and aberrant fibroblast activity^10^. In this study, we show that treatment of radiation wounds with plasminogen resulted in a correction of most of these events. Based on our results, we propose a working model for the role of plasminogen in the healing of radiation wounds (Fig. 5d). Radiation wounds have highly activated inflammation and tissue formation processes (Supplementary Table 2 and Figs. 2 and 3a–d, day 0), but starting from 1 day after plasminogen injection, the expression of many chemokines and interleukins responsible for the infiltration of immune cells decreases (Supplementary Table 3). At days 9 and 20, the signaling by the pro-inflammatory TNF and TLR pathways was significantly reduced by plasminogen, as was the expression of many pro-inflammatory cytokines (Supplementary Tables 3 and 4, and Fig. 3a–l). In addition, plasminogen induces a switch in the arachidonic acid pathway from pro-inflammatory prostaglandin synthesis to anti-inflammatory HETE and EET synthesis (Fig. 3m). All of these events facilitate the plasminogen-dependent termination of inflammation. Plasminogen also regulates formation of granulation tissue. At day 3 of treatment, plasminogen temporarily enhances fibroblast activation (as shown by enhanced expression of *Actc1*). However, from day 9, the signaling pathways regulating cell proliferation (TGF-β, WNT, and MAPK signaling) are downregulated by the plasminogen (Supplementary Tables 3 and 4, and Fig. 4a, c–k). In addition, plasminogen downregulates the expression of several growth factors and many proteins involved in angiogenesis and ECM remodeling. Together, these events result in the gradual resolution of granulation tissue, which is associated with a significant decrease in the expression of cell adhesion molecules (integrins and their receptors). Finally, plasminogen treatment also affects cell metabolism by decreasing glycolysis and increasing lipid synthesis (Supplementary Table 4 and Fig. 5b, c). High levels of glycolysis are characteristic for inflamed tissues^40,46^. By the time the re-epithelialization is completed in plasminogen-treated wounds, the inflammation and the tissue formation processes are fully resolved. In contrast, all these events are significantly delayed or abrogated in PBS-treated wounds.

Dermatological changes after irradiation often include fibrosis, and currently there is no standard protocol for the prevention or treatment of fibrosis^10,47^. Although it is known that TGF-β is the main regulator of fibrosis, preclinical attempts to systemically inhibit TGF-β or components of the TGF-β signaling pathway have failed due to serious side effects^37^. Here we show that local plasminogen treatment of radiation wounds results in downregulation of TGF-β signaling, and in decreased expression of many pro-fibrotic factors (Supplementary Tables 3 and 4, and Fig. 5a). Therefore, plasminogen might possibly be considered not only as a master regulator of wound healing, but also as an anti-fibrotic regulator.

In summary, we show here that plasminogen regulates the healing of radiation-induced wounds via a pleiotropic effect on the wound transcriptome. By “correcting” the expression of genes that are dysregulated in radiation wounds to levels that are in healthy skin, plasminogen leads to significantly enhanced wound healing without the risk for development of fibrosis. Therefore, we propose plasminogen as a promising drug candidate for treating radiation-induced wounds. However, an important question that needs to be addressed before initiation of clinical trials is whether local application of plasminogen decreases the anticancer effect of radiotherapy.

## Materials and methods

### Mice

The C57BL/6J wild-type mice were obtained from Scanbur (Karlslunde, Denmark). The age of the mice was between 12 and 16 weeks at the start of the experiments. The reason for using older mice was that most of the patients undergoing radiotherapy are at an older age and we wanted to mimic that. In addition, mice at the age of 6–8 weeks healed their radiation wounds much faster than older mice. The sex ratio in all experiments was evenly distributed between males and females. The animals were kept under standard laboratory conditions, and all experimental protocols were approved by the Regional Ethics Committee of Umeå University.

### Development of radiation-induced wounds

The dorsal skin of the mice was shaved 3 days prior to irradiation. For irradiation, the mice were anesthetized by intraperitoneal injection of 150 μl of a mixture containing 8% Ketaminol vet (REF. No Vnr 511485, Intervet AB, Sollentuna, Sweden) and 5% Dormitor vet (REF. No Vnr 015602, Orion Pharma AB, Espoo, Finland). The mice were then placed inside a lead box with 2-cm-thick walls to protect the whole body from irradiation, and the dorsal skin was gently stretched out through a 4-cm gap at the bottom of the box and affixed with medical tape, as described previously^6^. The lead box with the mouse was placed in a Gammacell 40 Exactor (Ashford, UK) that has two Cesium-137 sources, and radiation was given as a single dose of 1 Gy per minute over 20 min for a total dose of 20 Gy. After the irradiation, the mice were left untreated for 10 days to allow radiation wounds in the form of desquamation to develop (Fig. 1a).

### Plasminogen and PBS treatment

At day 10 after irradiation, designated here as day 0 of treatment, the mice were divided into two groups—one group was treated with plasminogen (human Glu-plasminogen in 137 mM NaCl, 2.7 mM KCl, 10 mM Na_2_HPO_4_, and 1.8 mM KH_2_PO_4_ defined as PBS buffer, Omnio AB, Umeå, Sweden) and the other was treated with PBS (placebo control). Plasminogen (100 µl of a 10 mg/ml solution) or PBS (100 µl) were administered daily as subcutaneous injections. For each injection, the mice were anesthetized using isoflurane. After 20 days of treatment, the mice were left untreated for another 10 days (Fig. 1a). The entire wound- healing process was documented via digital photographs taken every other day, and a ruler was used for standardization. The wound area was quantified from the photographs using ImageJ (National Institute of Health, Bethesda, USA). Skin samples for further examination were collected from mice euthanized by cervical dislocation at different time points. In the wound-healing study, six mice per group (treatment) have been used. Mice with very severe radiation wounds have been excluded.

### Morphological and immunohistochemical analyses

Skin from the wounded area taken at different time points was fixed in 4% paraformaldehyde, embedded in paraffin, and sectioned at a thickness of 6 µm perpendicular to the skin surface. For histological analyses, the sections were stained with Mayer’s hematoxylin (REF. No 01820, Histolab, Gothenburg, Sweden) using a standard protocol, and images were taken using a Leica DC300F digital camera attached to a Leica DM LB microscope (Leica, Wetzlar, Germany). Epidermal thickness was measured from the photographs using Adobe Photoshop. For each mouse, two sections were used, and three areas were quantified on each section. Mean values from these measurements are presented in figures. The skin sections (two sections for each mouse) were also examined blindly by a trained histopathologist and scored according to the scoring system shown in Supplementary Table 1. For all of the analyses, healthy non-radiated skin was used as the reference.

For immunostaining, sections were deparaffinized with xylene and rehydrated through washes in a graded ethanol series. Antigen retrieval was performed with citrate buffer at 95 °C, and the sections were blocked with serum-free protein block (REF. No X0909, DAKO, Carpinteria, CA, USA). Neutrophils were stained with the rat anti-mouse Ly-6B.2 monoclonal antibody (REF. No MCA771G, AbD Serotec, Oxford, UK) followed by biotinylated goat anti-rat IgG antibodies (REF. No sc-2041, Santa Cruz Biotechnology, Dallas, TX, USA) and streptavidin conjugated with Alexa Fluor 647 (REF. No S32357, Thermo Fisher Scientific, Waltham, MA, USA). Fibrin was stained with goat anti-mouse fibrin(ogen) antibody (Nordic Immunological Lab, Tilburg, the Netherlands) followed by rabbit anti-goat antibodies conjugated with Alexa Fluor 555 (REF. No A21431, Thermo Fisher Scientific). Proliferative cells were stained with rabbit antibody against mouse Ki67 protein (REF. No PA5-19462, Thermo Fisher Scientific, Waltham, USA) followed by DyLight 594-conjugated anti-rabbit IgG antibody (REF. No DI-1594, Vector Laboratories, Burlingame, USA). Vessels were stained with rat anti-mouse CD31 (cluster of differentiation 31 protein) antibody (REF. No 550274, BD Bioscience, San Jose, USA) followed by Texas Red conjugated goat anti-rat IgG antibody (REF. No sc-2782, Santa Cruz Biotechnology, Texas, USA). 8-oxo-2′-deoxyguanosine was stained with a goat polyclonal antibody (REF. No ab10802, Abcam, Cambridge, UK) followed by donkey anti-goat IgG HRP (REF. No ab97110, Abcam, Cambridge, UK) and DAB substrate (Cat No SK4100, Vector Laboratories, Burlingame, USA). Rabbit antibodies for mouse CTGF (REF. No ab6992, connective tissue growth factor), IL1β (REF. No ab9722), CD14 (REF. No ab203294), SPP1 (osteopontin) (REF. No ab8448), and TGF-β (REF. No ab92486) were obtained from Abcam, and antibody for FOSL1 (Fos-related antigen 1) (REF. No PA5-40361) was from Thermo Fisher Scientific. These antibodies were followed in immunostaining by Dylight 594-conjugated anti-rabbit IgG antibody (Vector Laboratories). TNF-α was stained with mouse monoclonal antibody (REF. No ab1793, Abcam) followed by anti-mouse antibody conjugated with Alexa Fluor 647 (Thermo Fisher Scientific). Finally, nuclei were stained with DAPI (REF. No 62248, Thermo Fisher Scientific), and images were captured with a Zeiss Axio Imager Z1 (Zeiss, Oberkochen, Germany). Quantification of fluorescent areas was performed using ImageJ software and had been done blindly by two persons. For all the immunohistochemistry studies, a minimum of three mice per time point and treatment were used. For each mouse, two sections were used, and three areas were quantified on each section. Mean values from these measurements are presented in figures.

### RT^2^ Profiler PCR arrays

Skin samples from the wounded area (100–200 mg) were cut into 1–2-mm^2^ pieces and kept in RNAlater (REF. No AM7020, Thermo Fisher Scientific) for 3 days at +4 °C. The samples were then homogenized in QIAzol (REF. No 1023539, Qiagen Sciences, Maryland 20874, USA) using Precellys CK28R tubes (REF. No KT03961-1007.2), on a Precellys 24 homogenizer (both from Bertin Technologies, Lyon, France) according to the manufacturer’s instructions. Total RNA was extracted with RNeasy Lipid Tissue Mini Kit (REF. No 1023539, QIAgen, Hilden, Germany) according to the manufacturer’s instructions. For the array study, four mice per time point and per treatment have been used. RNA samples from each treatment group and time point were pooled, and 5 µg of the pooled RNA was reverse-transcribed using a RT^2^ First Strand Kit (REF. No 330524, Qiagen Sciences, Maryland 20874, USA). Synthesized cDNA was immediately used for analysis or stored at −20 °C for up to 1 week. Gene expression was analyzed using a 96-well Mouse Wound Healing RT^2^ Profiler PCR Array Version 4.0 (REF. No PAMM-121Z) and Fibrosis Array (REF. No PAMM120z, both from Qiagen) according to the manufacturer’s instructions. For each treatment group and time point, the array was run three times. RT-PCR was run on a StepOnePlus™ Real-Time PCR system (Thermo Fisher Scientific). For data normalization, *Hsp90ab1* was selected as the reference gene based on data from the Mouse Housekeeping Genes RT² Profiler™ PCR Array (REF. No. 330231 PAMM-000ZA, Qiagen, Maryland, USA) and subsequent calculation of M values using the geNorm software (Biogazelle NV, Gent, Belgium). StepOne Software v.2.3 was used for data analyses, and gene expression was calculated with the ΔΔCt method. A gene was assumed to be differentially expressed if there was at least a twofold difference in the expression between different time points or genotypes, and the difference was statistically significant if *p* ≤ 0.05.

### mRNA sequencing and RT-PCR

Skin samples were incubated in RNAlater and homogenized in QIAzol (Qiagen Sciences, Maryland 20874, USA) using Precellys CK28R tubes (REF. No KT03961-1007.2), on a Precellys 24 homogenizer. Total RNA was extracted with RNeasy Lipid Tissue Mini Kit (REF. No 1023539, QIAgen, Hilden, Germany) according to the manufacturer’s instructions. The purity of the RNA from each individual mouse was checked with a NanoDrop 2000 spectrophotometer (Thermo Scientific), and the OD260/280 and OD260/230 were ≥2.0 for all the samples. The RNA quality was analyzed using a DNF-472 High Sensitivity RNA Analysis Kit, 15nt (REF. No. DNF-472-0500, Advanced Analytical, Santa Clara, USA) together with the Fragment Analyzer, and RNA-quality values were ≥6.8 for all of the samples. For this study, four mice per time point and per treatment have been used. RNA samples from each treatment group and time point were pooled, and 5 µg of the pooled RNA was sent to Novogene (Hong Kong) for transcriptome sequencing and data analysis. A gene was assumed to be differentially expressed if there was at least a twofold difference in the expression between different genotypes, and the corrected *p* value was ≤0.05.

For some genes, the expression was validated using quantitative real-time PCR with the comparative C_T_ method and with *TBP* (TATA-box-binding protein) as the internal reference gene. The selection of the reference gene was based on data from the RT² Profiler PCR Array Mouse Housekeeping Genes (REF. No PAMM 000Z, Qiagen) and subsequent calculation of M values using the geNorm software as described above. Gene-specific primers and probes (TaqMan Gene Expression Assays) were obtained from Applied Biosystems (REF. No. 4331182, Foster City, USA), and PCR mastermix 2× SsoAdvanced Universal Probes Supermix was from Bio-Rad (REF. No 172-5281, CA, USA). Each sample from individual mice was run in triplicate on the StepOnePlus Instrument (Applied Biosystems) using the real-time PCR conditions recommended for the 2× SsoAdvanced Universal Probes Supermix.

### Statistical analysis

All results are expressed as the mean ± SEM. In Fig. 1f, g, the differences between two groups were analyzed with the Mann–Whitney *U* test. For the rest of the figures, the differences between two groups were analyzed with two-tailed Student’s *t* tests. *P* ≤ 0.05 was considered statistically significant.

## Supplementary information

Supplemental figure legends

Supplemental figure 1

Supplemental figure 2

Supplemental figure 3

Supplemental figure 4

Supplemental figure 5

Supplemental figure 6

Supplemental figure 7

Supplemental figure 8

Supplemental figure 9

Supplemental figure 10

Supplemental figure 11

Supplemental table 1

Supplemental table 2

Supplemental table 3

Supplemental table 4
