# Supplementary material for: Plasminogen is a master regulator and a potential drug candidate for the healing of radiation wounds
Source: Cell Death Dis. 2020 Mar 23;11(3):201. doi: 10.1038/s41419-020-2397-0 (PMC7089956; doi:10.1038/s41419-020-2397-0)
Supplement: Supplementary file 1 — Supplemental figure legends [file 41419_2020_2397_MOESM1_ESM.docx]

**Supplementary Figure 1. Expression of selected plasminogen-regulated genes.** Expression of selected genes (as indicated) in radiation wounds before treatment (day 0) and at different time points of treatment with PBS or plasminogen as measured using RT-PCR. The data are presented as a fold change relative to mRNA levels in the control healthy, non-irradiated skin. **P < 0.05;* ***P < 0.01.*

**Supplementary Figure 2. Plasminogen treatment reduces reactive oxygen species (ROS) levels during the healing of radiation wounds.** (a - f) Representative photographs of 8-Oxo-2´-deoxyguanosine (brown) and Hematoxylin (purple) staining performed on paraffin sections of (a) control (no irradiation), (b) irradiated skin before treatment (day 0), (c and e) irradiated skin treated with PBS or (d and f) plasminogen at day 20 and 30 of the healing process. Scale bar = 100 µm.

**Supplementary Figure 3. Plasminogen treatment reduces the levels of IL1-β during the healing of radiation wounds.** (a - f) Representative photographs of IL1-β (red) staining performed on paraffin sections of (a) control (no irradiation), (b) irradiated skin before treatment (day 0), (c and e) irradiated skin treated with PBS or (d and f) plasminogen at day 20 and 30 of the healing process. DAPI staining is shown in blue. Scale bar = 100 µm.

**Supplementary Figure 4. Plasminogen treatment reduces the levels of SPP1 during the healing of radiation wounds.** (a - f) Representative photographs of SPP1 (red) staining performed on paraffin sections of (a) control (no irradiation), (b) irradiated skin before treatment (day 0), (c and e) irradiated skin treated with PBS or (d and f) plasminogen at day 20 and 30 of the healing process. DAPI staining is shown in blue. Scale bar = 100 µm.

**Supplementary Figure 5. Plasminogen treatment reduces the levels of TNF-α during the healing of radiation wounds.** (a - f) Representative photographs of TNF-α (red) staining performed on paraffin sections of (a) control (no irradiation), (b) irradiated skin before treatment (day 0), (c and e) irradiated skin treated with PBS or (d and f) plasminogen at day 20 and 30 of the healing process. DAPI staining is shown in blue. Scale bar = 100 µm.

**Supplementary Figure 6. Plasminogen treatment reduces CTGF (connective tissue growth factor) in radiation wounds.** (a - f) Representative photographs of CTGF (red) staining performed on paraffin sections of (a) control (no irradiation), (b) irradiated skin before treatment (day 0), (c and e) irradiated skin treated with PBS or (d and f) plasminogen at day 20 and 30 of the healing process. DAPI staining is shown in blue. Scale bar = 100 µm.

**Supplementary Figure 7. Plasminogen treatment reduces the levels of TGF-β during the healing of radiation wounds.** (a - f) Representative photographs of TGF-β (red) staining performed on paraffin sections of (a) control (no irradiation), (b) irradiated skin before treatment (day 0), (c and e) irradiated skin treated with PBS or (d and f) plasminogen at day 20 and 30 of the healing process. DAPI staining is shown in blue. Scale bar = 100 µm.

**Supplementary Figure 8. Plasminogen treatment reduces the levels of FOSL1 during the healing of radiation wounds.** (a - f) Representative photographs of FOSL1 (red) staining performed on paraffin sections of (a) control (no irradiation), (b) irradiated skin before treatment (day 0), (c and e) irradiated skin treated with PBS or (d and f) plasminogen at day 20 and 30 of the healing process. DAPI staining is shown in blue. Scale bar = 100 µm.

**Supplementary Figure 9. Plasminogen treatment reduces the levels of CD14 during the healing of radiation wounds.** (a - f) Representative photographs of CD14 (red) staining performed on paraffin sections of (a) control (no irradiation), (b) irradiated skin before treatment (day 0), (c and e) irradiated skin treated with PBS or (d and f) plasminogen at day 20 and 30 of the healing process. DAPI staining is shown in blue. Scale bar = 100 µm.

**Supplementary Figure 10. Plasminogen treatment reduces the number of proliferating cells (shown as Ki67 staining) in radiation wounds.** (a - f) Representative photographs of Ki67 (red) staining performed on paraffin sections of (a) control (no irradiation), (b) irradiated skin before treatment (day 0), (c and e) irradiated skin treated with PBS or (d and f) plasminogen at day 20 and 30 of the healing process. DAPI staining is shown in blue. Scale bar = 100 µm.

**Supplementary Figure 11. Plasminogen treatment reduces the amount of vessels (shown as CD31 staining) during the healing of radiation wounds.** (a - f) Representative photographs of CD31 (red) staining performed on paraffin sections of (a) control (no irradiation), (b) irradiated skin before treatment (day 0), (c and e) irradiated skin treated with PBS or (d and f) plasminogen at day 20 and 30 of the healing process. DAPI staining is shown in blue. Scale bar = 100 µm.

**Supplementary Table 1. Scoring system for healing of radiation-induced wounds.**

**Supplementary Table 2.** Gene expression in radiation wounds at day 10 after irradiation (just before treatment with plasminogen or PBS started), compared to normal healthy skin, measured using wound healing RT² Profiler PCR Array. For all genes, p ≤ 0.05.

**Supplementary Table 3.** Gene expression (shown as fold regulation) in plasminogen-treated radiation-induced wounds when compared to PBS-treated wounds, determined using wound healing and fibrosis RT² Profiler PCR Arrays. For all the genes, p ≤ 0.05. Genes that were deregulated in the wounds before plasminogen treatment (see Table 1) are marked with *, genes that are known to be involved in development of fibrosis are marked with #. Dash indicates a lack of statistically significant difference in gene expression, nd – not determined.

**Supplementary Table 4.** Gene expression at day 20 in plasminogen (plg)-treated radiation wounds when compared to PBS-treated wounds, or to healthy skin, studied using mRNA sequencing. All the genes had corrected p-value ≤ 0.05.
